# The Frequency of *Staphylococcus aureus* Isolated from Endocervix of Infertile Women in Northwest Iran 

**DOI:** 10.22074/ijfs.2016.4969

**Published:** 2016-11-11

**Authors:** Mohammad Taghi Akhi, Aylin Esmailkhani, Javid Sadeghi, Behrooz Niknafs, Laya Farzadi, Aydin Akhi, Elmira Najafi Nasab

**Affiliations:** 1 Immunology Research Center, Tabriz University of Medical Sciences, Tabriz, Iran; 2 Department of Bacteriology and Virology, School of Medicine, Tabriz University of Medical Sciences, Tabriz, Iran; 3 Department of Anatomical Sciences, Medical Faculty, Tabriz University of Medical Sciences, Tabriz, Iran; 4 Women’s Reproductive Health Research Center, Tabriz University of Medical Sciences, Tabriz, Iran; 5 Faculty of Pharmacy, Tabriz University of Medical Sciences, Tabriz, Iran

**Keywords:** Infertility, *S.taphylococcus aureus*, Endocervix, *mecA*

## Abstract

**Background:**

Infertility is one of the major social issues. Due to the asymptomatic cervical infection associated with *Staphylococcus aureus (S. aureus)*, the majority of patients remain undiagnosed. The present study intended to assess the frequency of *S. aureus* isolated from infertile women’s endocervix in northwest Iran.

**Materials and Methods:**

In a descriptive cross sectional study, specimens were randomly collected during vagina examination using a sterile speculum and swabbing. After performance of antibiotic susceptibility testing, polymerase chain reaction (PCR) was used to identify methicillin-resistance *S. aureus* (MRSA) and toxic shock syndrome toxin-1 (TSST-1).

**Results:**

About 26 (26%) and 9 (9%) women’s urogenital tracts were colonized by *S. aureus* and Candida spp., respectively, of which three (11.5%) patients were infected with fungi and *S. aureus*, simultaneously. Antibiotic susceptibility results showed high activity of vancomycin and co-trimoxazole on isolates. Regarding PCR results, mecA sequences were detected in 7 (26.9%) strains, whilst the tst gene encoding TSST-1 was not detected in any of clinical strains.

**Conclusion:**

The prevalence of *S. aureus* was very high in infertile women. Therefore, it demands all patients undergoing infertility treatment to be investigated thoroughly for this type of infection.

## Introduction

One of the most common reproductive health issues in developing countries is the high rate of infertility (1). It becomes a globally important subject for clinical research and practice because infertility affects 60 to 168 million individuals, both women and men, in reproductive age. It has been reported that of 10 couples, one couple incurs the early or secondary stages infertility (2, 3). Infertility is characterized as inability to conceive during one year, despite normal cohabitation (4), as indicated by the European Society of Human Reproduction and Embryology (ESHRE), which is evidenced in 10-15% of all couples (5).

There are a number of factors which are responsible for infertility in females. According to a study by Vayena et al. (3), the rates of primary infertility are generally between 1 and 8% with rates of secondary infertility reaching as high as 35%. Infection of reproductive organs is one of the most important factors affecting infertility, while the determination of the type of infection has a significant contribution to the treatment of this issue. However, the significance of these infections in the genital tracts is not well known. Many microorganisms, including bacteria, viruses, parasites and fungi, seem to be able to interfere with the reproductive function in both genders. Bacterial vaginosis is a prevalent issue among women with changing the balance of normal vaginal flora such as lactobacilli (6, 7). However, some related pathogenic impacts have been evidenced such as increased rates of premature rupture of the membranes (PROM), late miscarriage in first trimester, preterm labor, endometriosis and delivery (6). Bacterial vaginosis is the most common lower genital tract disorder among reproductive age of women (pregnant and nonpregnant) and the most common cause of vaginal odor and malodorous discharge (8). 

The bacteria encountered in the female genital tract can be divided into aerobic and anaerobic organisms. Among the aerobic Gram-positive organisms, there are several species of *Streptococcus (S.)*, such as group B S. (GBS), and among Gram-positive facultative anaerobic organisms, there are several species of *Enterococcus (E.)* (9). *S. aureus* is an infrequent but one of the most successful human pathogens. It has the ability to cause a number of infections in various environmental corners within the host. *S. aureus* has additionally been reported to be commonly isolated microorganism from cervical specimens (10). It is found in the genital tract of approximately 9 to 10% of asymptomatic women, approximately 10% of patients with postoperative wound abscess after gynecologic or obstetric procedures, and 5 to 20% of genital tract cultures of women with pelvic infection. It is also detected in virtually 100% of women who have toxic shock syndrome toxin-1 (TSST-1) (9). This study was, therefore, designed to assess the frequency of *S. aureus* isolated from infertile women’s endocervix in northwest Iran. 

## Materials and Methods

### Sampling

Descriptive cross sectional study was carried out at the Tabriz University of Medical Science (TUMS), Tabriz, Iran, between April and July 2015. The cervical samples were randomly taken from 100 women who attended the Department of Obstetrics and Gynecology of Milad Infertility Center affiliated to TUMS for unexplained infertility during the mentioned-period. Patients with infertility due to unrelated reasons to *S. aureus* infections, such as ovarian cancer, lazy ovary, ovarian cysts and polycystic ovary syndrome (PCOS), were excluded from the study. 

The Ethic Commission of TUMS approved this study (Number: 5/412912-2015). After obtaining a written consent form from all patients, the specimens were collected using a sterile vaginal speculum and swab. 

### Isolation and identification

Samples were streaked on blood agar and mannitol salt agar (Merck, Germany) plates and incubated aerobically at 37°C for 24-48 hours. After overnight incubation, the isolates were examined by Gram staining technique using catalase, DNase and coagulase tests (11).

### Antibiotic susceptibility testing 

The susceptibility of *S. aureus* isolates to antimicrobial agents was measured *in vitro* using the disc diffusion method according to Clinical and Laboratory Standards Institute (CLSI) protocols (12). The tested antibiotics included penicillin, gentamicin, ciprofloxacin, vancomycin, trimethoprim/sulfamethoxazole and cefoxitin (MAST Diagnostics, UK). 

### Detection of mecA and tst genes

Bacterial DNA was extracted from the isolates according to tissue buffer boiling method (13). Firstly, 20 µl tissue buffer [0.25% sodium dodecyl sulfate (SDS)+0.05M NaOH (CinnaClon, Iran)] were mixed with one colony of bacterial isolate, the combination was incubated for ten minutes in 95°C, the mixture was centrifuged for one minute in 13000 g, 180 µl Milli-Q water (CinnaClon, Iran) were slowly added, and finally extracted DNA was frozen in -20°C for durable storage. 

DNA isolates with the concentration of 0.1 ng/µl were used as the templates for polymerase chain reaction (PCR) analysis. Multiplex PCR was carried out by CinnaGen MsterMix (CinnaClon, Iran) using the mecA and tst primers, as described previously (14). 

The sequences of the mecA primers used were:

5´-ACTGCTATCCACCCTCAAAC-3´ and 5´- CTGGTGAAGTTGTAATCTGG -3´, 

while the sequences of the tst primers used were: 

5´-ACCCCTGTTCCCTTATCATC-3´ and 5´-TTTTCAGTATTTGTAACGCC-3´ (synthesized at the CinnaClon, Iran). 

The strains 92-S-1344 (tst) and 95-S-739 (mecA) were used as positive control in this study. Amplification was carried out using a thermocycler (Eppendorf, Germany) as follows: i. Initial denaturation at 94°C for 5 minutes, ii. 35 cycles of denaturation at 94°C for 2 minutes, annealing at 57°C for 2 minutes, and primer extension at 72°C for 1 minutes, and iii. Terminal extension at 72°C for 7 minutes. Electrophoresis of PCR products was performed on 1% agarose gel (CinnaClon, Iran). The gel staining was performed in ethidium bromide for 20 minutes and visualized using a gel documentation system (UVP, USA). 

## Results

During the study period, a total of 100 infertile women were included in this study. All participants underwent intrauterine insemination (IUI). About 26 (26%) and 9 (9%) women’s urogenital tracts were colonized by *S. aureus* and Candida spp., respectively, which were identified by mycology methods. Among them, three (8.5%) patients were infected with fungus and *S. aureus*, simultaneously. The average age of these patients was 30.96 years, ranging between 18 and 56 years. Of the patients who were colonized by *S. aureus*, 11 (42.1%) were under 30 years and 14 (53.7%) were over 30 years of age. After examining the wives of the patients who were colonized by *S. aureus*, the semen samples of four (15.38%) couples were positive for *S.aureus*. In addition, penicillin-resistant strains of *S. aureus* were colonized in the reproductive system of a couple. Of those *Candida* spp. carriers, three (3 of 9) wives were also infected with Candida spp. The results of antibiotic susceptibility testing are summarized in Table 1. 

**Table 1:** The results of antibiotic susceptibility testing


Antibiotic	Sensitive (%)	Intermediate (%)	Resistant (%)

Penicillin	3 (11.53)	0 (0)	23 (88.46)
Gentamicin	19 (73.07)	0 (0)	7 (26.09)
Ciprofloxacin	19 (73.07)	0 (0)	7 (26.09)
Vancomycin	25 (96.15)	1 (3.84)	0 (0)
Co-trimoxazole	25 (96.15)	0 (0)	1 (3.84)
Cefoxitin	19 (73.07)	0 (0)	7 (26.09)


The data were presented as N (%).

In general, vancomycin and co-trimoxazole showed high activity against the isolates. Regarding PCR results, *mecA* sequences were detected in 7 (26.9%) isolates, whilst the tst gene encoding TSST-1 was not detected in any of clinical isolates.

**Fig.1:**
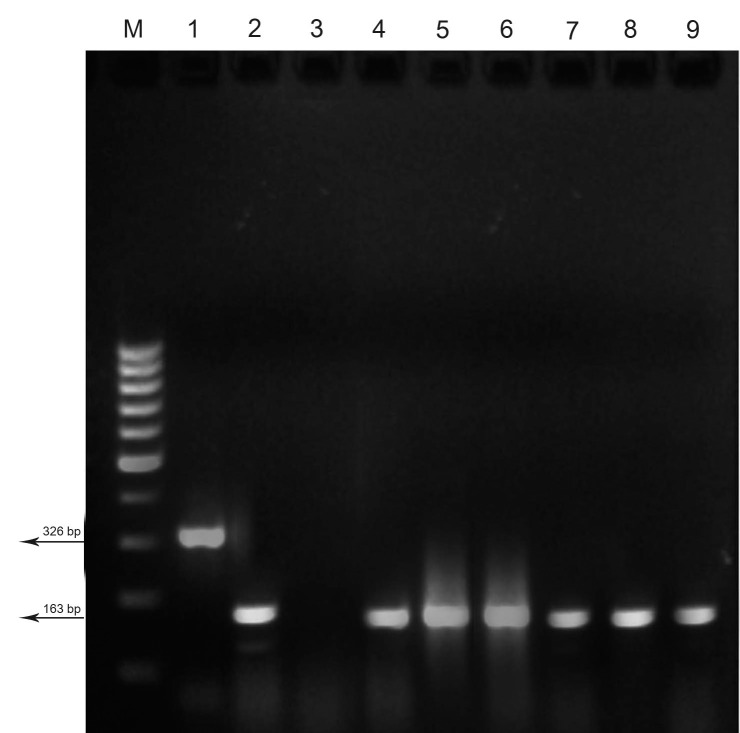
Multiplex PCR product for the mecA and tst genes. M; 100 bp molecular weight marker, Lane 1; tst gene positive control (ATCC 92-S-1344), Lane 2; mecA gene positive control (ATCC 95- S-739), Lane 3; Negative control, and Lane 4-9; mecA positive .

## Discussion

The study investigated the prevalence of *S. aureus* in infertile women. An in-depth study on these genera has not yet been conducted in Iran. Generally, infectious vaginitis is a prevalent disorder with noteworthy clinical results if left untreated. Infertility is an important health issue with far-reaching consequences on couple, family planning program, health system and spread of sexually transmitted diseases (STD) and acquired immunodeficiency syndrome (AIDS). It can be characterized as the lack of a conception after at least one year of constant, unprotected sexual intercourse (15).

In a study by Okonofua et al. (10), *Candida albicans* (25%), *S. aureus* (21.7%) and *Neisseria gonorrhoeae* (17.4%) were the most commonly isolated microorganisms; however, there was no difference between fertile and infertile women in the rates of isolation of these pathogens. In most cases, resistance to penicillin is attributable to β-lactamase production. We found that the most antibiotic resistance was against penicillin, which is not supported by a study conducted by Ghiasi et al. (16), in which they have indicated 100% of *S. aureus* were sensitive. Our findings also indicated that none of the *S. aureus* strains were resistant to vancomycin. In a study by El-Ghodban et al. (17), they have reported that less than 50% of *S. aureus* strains were β-lactamase producers and resistant to penicillin. However, almost 75% of the strains originating from food were positive for β-lactamase and resistant to penicillin. In our study, all isolates were positive for *mecA* genes, while they were resistant to the gentamicin, ciprofloxacin and cefoxitin. de A Trindade et al. (18) have also reported that among methicillin-resistant *S. aureus* (MRSA) isolated from blood samples, twenty (13%) individuals were susceptible to four or more antimicrobials.

The incidence of TSST-1-producing strains has been registered worldwide (19). Colonization with *S. aureus* is generally highest (20 to 30%) in the oropharynx or nose of non-healthcare workers. Vaginal colonization with *S. aureus* has been determined to be lower (10 to 20%) in the United States, Europe, and Asia (20). Similarly, TSST- 1-producing strains of *S. aureus* have been isolated vaginally from 1 to 4% of healthy, menstruating women in the general population (19). Due to a higher immune response to TSST-1, S. infections are more common in developing countries than developed countries (21). We decided to use tst in the present study because of limited reports from many developing countries. In a study by Parsonnet et al. (19) carried out on Japanese women, of the 159 *S. aureus* isolates recovered, 14 (9%) were TSST-1 positive, suggesting that only 47% of women had positive titers of anti- TSST-1 antibody, which is significantly lower than the reported seropositivity rates in the Europe and United States (20). In the same study by El-Ghodban et al. (17), TSST-1 was detected in only three (7.5%) of 40 *S. aureus* clinical strains and in none of the food strains. In another study by Tsen et al. (22), they have firmly reported the comparative discoveries, in which only three (4.8%) of 62 strains of *S. aureus* from clinical sources as tst-carrying strains were identified using PCR, but none of their food strains carried this gene.

In the etiologies of infertility, the most contributed factors are related to female (40 to 55%) followed by male factors (30 to 40%), both partners (10%) and unexplained factors (10%) (1). There are several factors which increase risks for acquisition of bacterial vaginosis, while bacterial vaginosis is more prevalent in women who smoke (23), black women (24), women who are sexually active compared with virginal women (25), and women who utilize vaginal douches (26). 

The infertility leads to decreased levels of personal well-being, while for many individuals, it causes more serious consequences (27). Subsequently, it appears that screening is a reasonable approach that is likely to be cost effective. However, all physicians must have a high index of suspicion and utilize promptly accessible screening methods to detect and treat the patients with infectious vaginitis adequately (28). A limitation of the present study was the lack of evaluation of *S. aureus *in fertile women. However, it is suggested that further studies will be conducted on a larger sample.

## Conclusion

The exact knowledge of *S. aureus* colonization rate in infertile women has a great importance. Therefore, it demands all patients undergoing infertility treatment to be investigated thoroughly. Such screening and treatment during the course of infertility treatment increase the pregnancy rate extensively. However, randomized studies with larger number of participants are needed to reach more validated conclusions. 
